# H2B Type 1-K Accumulates in Senescent Fibroblasts with Persistent DNA Damage along with Methylated and Phosphorylated Forms of HMGA1

**DOI:** 10.3390/proteomes9020030

**Published:** 2021-06-21

**Authors:** Kévin Contrepois, Carl Mann, François Fenaille

**Affiliations:** 1 Institute for Integrative Biology of the Cell (I2BC), Université Paris-Saclay, CEA, CNRS, 91190 Gif-sur-Yvette, France; kcontrep@stanford.edu; 2 Department of Genetics, Stanford University School of Medicine, Stanford, CA 94305, USA; 3 Stanford Cardiovascular Institute, Stanford University, Stanford, CA 94305, USA; 4 Département Médicaments et Technologies Pour la Santé (DMTS), Université Paris-Saclay, CEA, INRAE, MetaboHUB, 91191 Gif-sur-Yvette, France

**Keywords:** histone variants, histone post-translational modifications, mass spectrometry, senescence, quiescence

## Abstract

Cellular senescence is a state of terminal proliferative arrest that plays key roles in aging by preventing stem cell renewal and by inducing the expression of a series of inflammatory factors including many secreted proteins with paracrine effects. The in vivo identification of senescent cells is difficult due to the absence of universal biomarkers. Chromatin modifications are key aspects of the senescence transition and may provide novel biomarkers. We used a combined protein profiling and bottom-up mass spectrometry approach to characterize the isoforms and post-translational modifications of chromatin proteins over time in post-mitotic human fibroblasts in vitro. We show that the H2B type 1-K variant is specifically enriched in deep senescent cells with persistent DNA damage. This accumulation was not observed in quiescent cells or in cells induced into senescence without DNA damage by expression of the RAF kinase. Similarly, HMGA1a di-methylated and HMGA1b tri-phosphorylated forms accumulated exclusively in the chromatin of cells in deep senescent conditions with persistent DNA damage. H2B type 1-K and modified HMGA1 may thus represent novel biomarkers of senescent cells containing persistent DNA damage.

## 1. Introduction

Cellular senescence is a stress response of mammalian cells characterized by a stable cell cycle arrest despite remaining metabolically active. Senescence can be induced by diverse stimuli including telomere loss (which results from repeated cell divisions), oncogene activation, and genotoxic agents [1]. Regardless of the stress, senescent cell cycle arrest is mediated by p53 and/or Rb tumor suppressor pathways. Moreover, they often display an enlarged and flattened morphology with increased expression of SA-β-galactosidase, secretion of some cytokines and metalloproteases, and profound chromatin reorganization (i.e., heterochromatin assembly) that may include the formation of highly compacted DNA in the form of senescent-associated heterochromatic foci (SAHFs) [1,2]. Accumulating evidence shows that cellular senescence plays critical roles in tumor suppression, wound healing, and aging in vivo [2].

Chromatin is the heritable material in eukaryotes and is composed of DNA, histone, and non-histone proteins. The building block of chromatin is the nucleosome that is composed of pairs of core histones H2A, H2B, H3, and H4. As the primary component of chromatin, histone post-translational modifications have been implicated in virtually all cellular processes requiring access to the genome by modulating local chromatin organization (i.e., transcription, DNA replication, and repair) [3,4,5,6]. Histone functions are regulated by a myriad of chemical modifications including acetylation, methylation, and phosphorylation. For example, H3-K4Me3 has been linked to transcriptional activation by rendering chromatin permissive to the transcriptional machinery (i.e., euchromatin). On the contrary, H3-K9Me2/3 marks are associated with transcriptional silencing by heterochromatin assembly. Histones H2A and H2B are less extensively modified than H3, but instead, are present as multiple variants that slightly differ in amino acid sequence. In humans, there are about 12 H2A variants [7], 16 H2B variants [8], and 5 H3 variants, according to the HISTome2 database [9]. H4 was thought to be a unique species until the recent discovery of a second isoform [10]. Several of these variants have been extensively studied for their crucial roles in transcriptional silencing (macro-H2A), activation (H2A.Z), and DNA repair (H2A.X) [11]. However, nuclear functions of other H2A and H2B variants remain to be determined.

Histones are mainly encoded by a family of replication-dependent genes located at two genomic clusters (cluster 1 on chromosome 6p22 and cluster 2 on chromosome 1q21). Histone mRNAs are the only known cellular mRNAs that end by a stem-loop instead of a polyadenylated tail [12]. This uncommon structure is necessary to confer a short half-life to these mRNAs when compared to polyA mRNAs. In fact, during the relatively short replication phase, newly synthetized DNA needs to be rapidly packaged with new and old histones. Hence, the S-phase is accompanied by an approximately 30-fold induction of histone mRNAs. When replication is completed, histone mRNA levels need to quickly diminish in order to avoid a toxic accumulation of histone proteins. Several histone variants are polyadenylated and expressed throughout the cell cycle (replication-independent genes) such as H3.3, H2A.J, and H2A.Z [11].

The stem-loop consists of a 6-base stem and a 4-nucleotide loop recognized by the stem-loop binding protein (SLBP). This protein is highly expressed during the S-phase and is crucial for mRNA 3′ processing, nuclear export, and translation [12]. In contrast, SLBP is present at very low levels in non-proliferating cells (differentiated, quiescent, senescent cells). Canonical histone mRNAs are rapidly degraded in the absence of SLBP unless there is a polyadenylation site downstream of the stem-loop sequence to stabilize the transcript [13,14,15,16].

In proliferating cells, chromatin maintenance is intrinsically linked to DNA replication by activation of histone mRNA transcription and facilitated deposition of histones by histone chaperones such as CAF1 [4,17]. However, non-proliferating senescent cells do not undergo S-phase chromatin assembly but utilize replication-independent chromatin assembly pathways to maintain chromatin structure and dynamics (for example, histone chaperones HIRA [18], DAXX/ATRX [19], and the DEK complex [20]). The functional consequences of these pathways in chromatin composition and dynamics during long-term cell cycle arrest remain to be explored. Senescent cells can persist in the body for decades (i.e., benign human nevi), but the state of senescent chromatin over long periods is only partially understood.

In this study, we characterized chromatin composition in various senescent and quiescent states. We used a combined protein-profiling (top-down) and bottom-up approach using mass spectrometry [21] to analyze all main core histone and high mobility group A (HMGA) post-translational modifications (PTMs) and variants in these conditions. Using this methodology, we previously described the accumulation of the H2A.J variant in fibroblasts induced into senescence by DNA damage, and the accumulation of H2A type 1-C in both quiescent and senescent cells [22]. Here, we investigated the dynamics of other core histones and associated variants as well as some specific abundant PTMs in relation to the senescence phenotype. Thus, we investigated H2B isoforms and showed that the H2B type 1-K histone variant markedly accumulated in fibroblasts induced into senescence by DNA damage by a post-transcriptional regulation after accumulation of the polyA mRNA forms.

H4-K20Me3 is a repressive mark reported to increase in senescent cells that participates in transcriptional repression and chromatin compaction [23,24]. H3-K27methylation is a repressive mark associated with facultative heterochromatin [25], whereas H3-K36 methylation is associated with multiple functions [26]. We observed that H4-K20Me3, H3.1/2-K27Me2/Me3, and H3.1/2-K36Me2 accumulated with time during quiescent and senescent proliferative arrests.

The HMGA1/2 proteins accumulate in senescent fibroblasts and contribute to chromatin compaction [27,28]. We analyzed HMGA1 PTMs in long-term cell cycle arrest states including multiple early (5 days) and deep (20 days) senescent (oncogene activation, genotoxic stress, telomere attrition) and quiescent conditions. We found that HMGA1 proteins are overexpressed in senescent cells and underwent senescent-specific modifications: HMGA1a di-methylation and HMGA1b tri-phosphorylation increased in deep senescent conditions. Our results reveal characteristic modifications of the chromatin that are shared in several non-proliferative states or are specific to senescent cells.

## 2. Materials and Methods

### 2.1. Cell Lines and Retroviruses

WI-38hTERT human embryonic fibroblasts expressing a conditionally activated form of the RAF1 kinase (GFP-RAF-ER) were cultured as described [29]. WI-38 cells were passaged in ambient 20% oxygen and 5% CO_2_ to obtain an early replicatively senescent population at population doubling (PD) 65. These cells were further maintained in culture for an additional month to obtain a deep replicatively senescent population at PD 66. MRC-5 human lung fibroblasts were cultured in a similar fashion to obtain a deep replicatively senescent population. Retroviral preparations of pBabe, pBabe-TRF2Δ (dominant-negative lacking the basic and Myb domains) were prepared as described [27,30].

### 2.2. Chemicals

Chemicals were prepared as 1000× stock solutions in the indicated solvents and stored at −20 °C. 4-hydroxy-tamoxifen (4-HT, Sigma H6278, Sigma-Aldrich, Saint Quentin Fallavier, France) was dissolved in ethanol. Etoposide (Sigma E1383, Sigma-Aldrich, Saint Quentin Fallavier, France) was solubilized in DMSO.

### 2.3. Preparation of Histones and HMGA Proteins, and Mass Spectrometry Analyses

Histones and HMGA proteins were acid-extracted and analyzed by MS and MS/MS both at the intact protein and tryptic peptide levels as previously described [21]. Profiling was performed by UHPLC-MS using an LTQ-Orbitrap mass spectrometer (ThermoFisher Scientific, Les Ulis, France) operating in the positive ion mode at a 30,000 resolution. Proteins were identified by their accurate mass measurement after deconvolution using the Xtract software (ThermoFisher Scientific). For tryptic peptide analyses, histones were first propionylated on lysine residues, then digested with trypsin, and finally, subjected to a second round of propionylation to block the newly formed N-terminal residues [31]. Analyses were then performed on a LTQ-Orbitrap Discovery mass spectrometer that was operated in the data-dependent acquisition mode, allowing the automatic switching between MS and MS/MS. The MS survey scan was performed from *m/z* 300–2000 in the Orbitrap, using a resolution set at 30,000 (at *m/z* 400). The five most abundant ions (threshold 500 counts, charge states higher than +1) were further selected for collision-induced dissociation (CID) experiments. The CID mass spectra were collected in the linear ion trap.

Relative quantification of deconvoluted intact protein modified forms/variants was performed by dividing the intensity of a given deconvoluted MS peak by the sum of the intensities of the different deconvoluted MS peaks composing the spectrum of a considered protein. Modified peptide sequences were first manually searched in the MS trace and then confirmed by visual inspection and interpretation of the corresponding MS/MS spectra. Relative quantification of PTMs was performed by measuring the area of the extracted ion chromatogram peak corresponding to a specific modified peptide normalized to the sum of the peak areas corresponding to all observed modified and non-modified forms of this peptide. In addition, relative quantification of histone variants was realized by measuring the area of the extracted ion chromatogram peak corresponding to a variant-specific peptide normalized to a peptide found in all corresponding variants.

### 2.4. Flow Cytometry Analyses of DNA Content

DNA content analysis was performed with a FACS Calibur flow cytometer (BD Biosciences, le Pont de Claix, France) essentially as described [29].

### 2.5. BrdU Incorporation and Immunostaining

Cells were seeded in 24 well plates at a density of 50,000 cells/well on collagen-treated coverslips. BrdU was added to media at a final concentration of 50 μM for 24 h. Immunofluorescence to visualize incorporated BrdU and γH2AX foci was performed as described [28]. DAPI CV (coefficient of variation) measurements were realized using an ImageJ in for semi-automatic quantification of DNA compaction as previously described [28].

### 2.6. qRT-PCR

Histone variants mRNA quantities were assessed by qRT-PCR. 500,000 cells were harvested in each condition prior to total RNA isolation using a Nucleospin RNA XS kit (Macherey-Nagel). Reverse transcription was performed using random hexamer and oligo(dT)18 primers. Variant-specific histone primers were designed to quantify the total mRNA level and the PolyA subpopulation (listed in Table S1). Quantitative real-time PCR was performed on a Bio-Rad iQ5 instrument. The reactions were prepared using Platinum SYBR Green qPCR SuperMix-UDG (Invitrogen 11733-046). GAPDH was used as a control gene for normalization.

### 2.7. Data Analysis and Statistics

The percentage of BrdU-positive cells was determined by counting at least 200 cells. γH2AX foci were counted in at least 100 nuclei in each condition. The DAPI CV was calculated for the indicated number of nuclei. DAPI CV results are presented as boxplots. A box represents 50% of the data and the median. Whiskers correspond to the minimum and maximum values. Histone PTM and variant relative abundances were presented on stacked histograms as the average values and standard deviations for the indicated number of biological replicates.

## 3. Results

**Figure 1:**
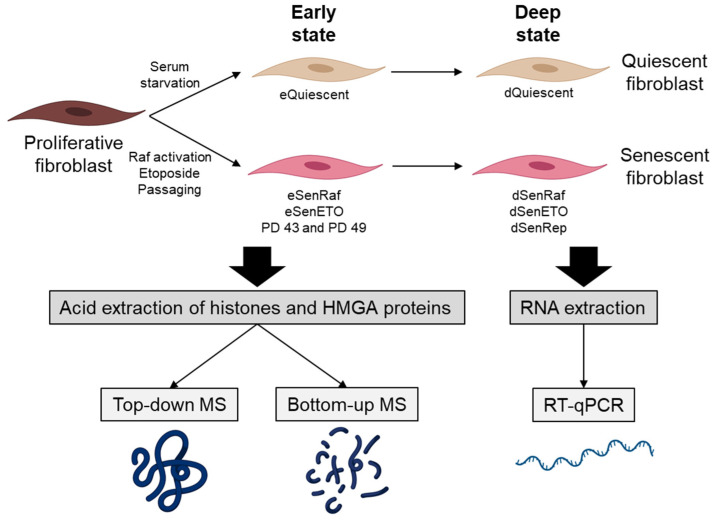
Overview of the study. WI-38 human fibroblasts were induced into quiescence by serum starvation or into senescence following Raf activation (SenRaf), etoposide treatment (SenETO), and passaging (Replicative senescence, SenRep). These various states of cell arrest were maintained for 5 days (early state) or 20 days (late state) and histones and HMGA were analyzed by mass spectrometry and RNA by RT-qPCR. Each condition was analyzed in triplicates except replicative senescence in duplicate. PD: population doubling.

In this study, we were interested in analyzing major core histone and HMGA PTMs and variants in early (5 days after treatment) and deep (20 days after treatment) senescent states. Our reference population was WI-38hTERT human embryonic lung fibroblasts grown in 5% oxygen. These cells were immortalized by expression of the telomerase to prevent stress engendered by telomere attrition or growth under hyper-physiological 20% ambient oxygen. They also expressed a fusion protein composed of a constitutively active form of the RAF1 kinase fused to GFP and the estrogen receptor domain (GFP-RAF-ER). The estrogen receptor domain is sequestered in an inactive form that can be activated by the addition of the ER ligand 4-hydroxy-tamoxifen (4-HT). Activation of the RAF1 kinase leads to a rapid hyper-stimulation of the MAP kinase pathway that induces senescence within 3 days [29]. Senescence was induced by (i) oncogene RAF activation for 5 days (eSenRAF) and 20 days (dSenRAF), (ii) genotoxic stress by treating cells with etoposide (a topoisomerase 2 inhibitor) (5 days: eSenETO and 20 days: dSenETO), (iii) telomere erosion (i.e., replicative senescence) (eSenRep, dSenRep). These conditions were compared to proliferating and quiescent serum-starved WI-38 fibroblasts (5 days: eQuiescent, 20 days: dQuiescent) (Figure 1).

All senescent conditions presented some level of chromatin compaction as observed by quantifying the coefficient of variance of the DAPI staining of DNA (Figure S1A) and cell cycle arrest was confirmed by BrdU incorporation and flow cytometry (Figure S1B). Histone and HMGA proteins were analyzed in each condition by mass spectrometry using a combined protein-profiling and bottom-up approach to characterize the main PTMs and variants at the protein and peptide levels, respectively [21]. The protein profiling performed by UHPLC-MS using a high-resolution high-mass accuracy Orbitrap instrument allowed the convenient detection and identification of the main histone post-translational modifications and variants thanks to a mass accuracy better than 10 ppm [21]. Distinction of post-translational modifications with the same nominal mass, such as acetylation and trimethylation (42.01 and 42.05 Da, respectively) cannot be accurately performed at the intact protein level. However, the bottom-up approach makes it possible first thanks to high mass accuracy measurement and high-resolution, and then by their respective MS/MS spectra. Moreover, acetylated and trimethylated peptides elute at different retention times, thus providing an additional identification criterion [32,33]. Hence, post-translationally modified residues and variant-specific peptides from core histones were identified by bottom-up proteomics with trypsin digestion and including 2 rounds of propionylation (pre- and post-digestion) [21]. RNAs were also analyzed to gain regulatory insights.

The early senescent samples (5 days) show very few differences in the relative abundance of histone PTMs and variants despite cell cycle arrest and heterochromatin assembly (Figure S1). However, striking differences were observed in deep senescent conditions when cells were kept in culture for 20 days (see below).

### 3.1. H2B type1-K Is Specifically Enriched in Deep Senescent Conditions with Persistent DNA Damage by an Active Post-Transcriptional Regulation

**Figure 2:**
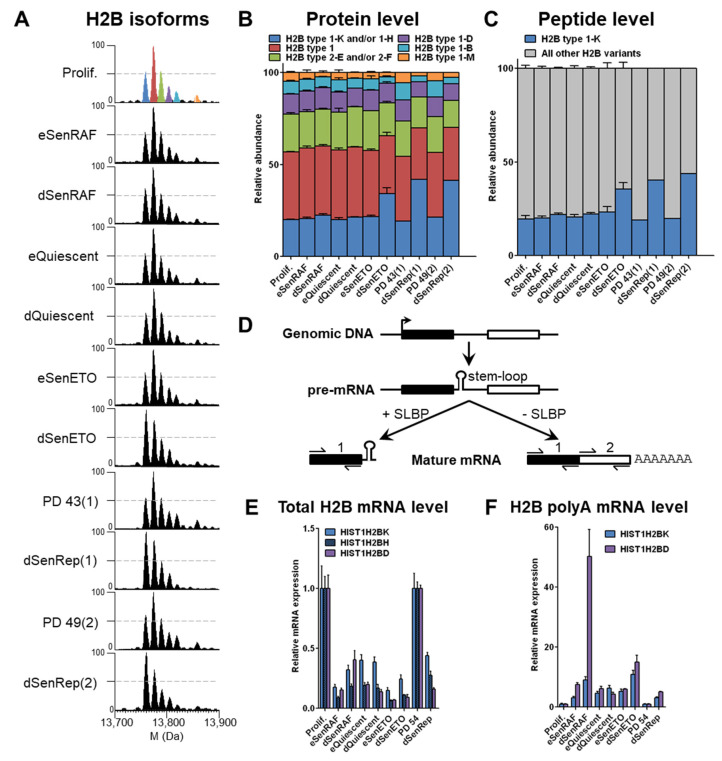
H2B type 1-K is specifically enriched in deep senescent cells with persistent DNA damage by an active post-transcriptional regulation. (**A**) Deconvoluted mass spectra of histone H2B; 50% intensity is represented by a dashed bar. Blue peak = H2B type 1-K and/or 1-H; Red peak = H2B type 1; Green peak = H2B type 2-E and/or 2-F; Purple peak = H2B type 1-D; Sky blue peak = H2B type 1-B; Orange peak = H2B type 1-M. Relative abundance of H2B isoforms quantified at the (**B**) protein and (**C**) peptide levels. Intact proteins were identified by accurate measurement of monoisotopic masses (Table S2). Peptide Leu100-Lys125 is specific to H2B type 1-K and corresponding MS/MS spectra are given in Figure S3. Experiments were performed on three independent biological replicates. (**D**) Processing of histone mRNA when SLBP is present (cell proliferation) or not (cell cycle arrest). To quantify total and polyA mRNA levels, primers were designed upstream of the stem-loop (1) and downstream the stem-loop (2), respectively. qPCR quantification on randomly reverse-transcribed cDNA of (**E**) total and (**F**) polyA mRNA levels of H2B variants. *HIST1H2BK*, *HIST1H2BH*, and *HIST1H2BD* genes encode for H2B type 1-K, H2B type 1-H, and H2B type 1-D proteins, respectively.

Histone H2B is predominantly unmodified and is present in 6 main peaks corresponding to potentially 8 variants based on accurate measurement of intact protein masses (within 10 ppm, Table S2). In early senescent conditions, no significant difference in the relative abundance of histone H2B variants was observed (Figure 2A,B). However, a reproducible relative increase of the “blue” peak, corresponding to H2B type 1-K alone or in combination with H2B type 1-H, since both isotope massifs might overlap to some extent (based on intact protein masses, Table S2), was observed in conditions of deep senescence with persistent DNA damages, i.e., dSenETO and dSenRep, as indicated by the large number of γH2AX foci (Figure 2 and Figure S1C). The relative abundance of the corresponding “blue” peak increased by 2-fold in these conditions to reach almost 40% when compared to other conditions. An increase of this peak was also visible in WI-38 fibroblasts expressing a dominant negative form of TRF2 that induces chromosome deprotection and senescence (dSenTRF2D) as well as replicatively senescent human lung MRC-5 fibroblasts (MRC-5 dSenRep) (Figure S2). This observation seems to indicate that an active phenomenon is responsible for the relative enrichment of specific H2B variants in deep senescent populations induced by DNA damage. Despite the high sequence similarity of H2B variants (Table S3), H2B type 1-K differs from the others by a Ser124Ala substitution, which results in 2 Da- or >16Da-mass differences when compared to H2B type 1-H or other H2B variants, respectively (Table S2).

The presence of H2B type 1-K was further ascertained by monitoring the corresponding specific peptide (Figure S3). Relative quantification of the abundance of tryptic C-terminal peptides Leu100-Lys125 unambiguously identified H2B type 1-K as the specific H2B variant that increased in deep senescence with DNA damage (Figure 2C). Altogether these data confirmed attribution of the “blue” peak to H2B type 1-K, while also showing the specific accumulation of this variant in deep senescent conditions with persistent DNA damages. This increase can be due either to a transcriptional regulation (increased mRNA levels) or a post-transcriptional regulation (increased translation, deposition and/or decreased degradation, eviction).

To investigate the mechanisms involved in this regulation, we performed qPCR experiments on randomly primed reverse-transcribed cDNA using 2 pairs of primers that hybridize to: (i) the mRNA 5′ upstream of the stem-loop that gives the total mRNA level and (ii) the mRNA 3′ downstream of the stem-loop and before the polyadenylation site that gives the polyA mRNA level (Figure 2D). We used the *HIST1H2BD* gene as an internal control that coded for an H2B variant that remained globally constant at the protein level in all conditions. As anticipated, the total mRNA of all tested H2B variants was decreased by 3–10 fold in quiescent and senescent proliferative arrest conditions reflecting the instability of the predominant stem-loop RNAs in these conditions [13,14,15,16] (Figure 2E). However, we found that *HIST1H2BK* (encoding H2B type 1-K) and *HIST1H2BD* (encoding H2B type 1-D) polyA mRNAs were both enriched 5–20 fold in all arrested conditions compared to the proliferating control (Figure 2F). Thus, a basal expression of H2B genes with polyadenylation sites explains the enrichment of polyA H2B mRNAs when SLBP is absent in conditions of long-term cell cycle arrest. Strikingly, H2B type 1-K protein is enriched to a greater extent than H2B type 1-D (Figure 2A,B), even though polyadenylated *HIST1H2BD* RNA is equal or greater than *HIST1H2BK* polyadenylated RNA in senescence (Figure 2E). This observation suggests that the enrichment of H2B type 1-K protein must involve some post-transcriptional mechanism (i.e., increased export, translation, deposition efficiencies or decreased eviction, degradation).

### 3.2. H4 Mono-Acetylation Remained Low in Deep Senescent States and H4-K20Me3 Increased Progressively with Time in Conditions of Cell Cycle Arrests

In a previous study [28], we described a specific decrease of 25% of H4 mono-acetylation localized at K16 by the NAD+-dependent deacetylase SIRT2 in early senescent conditions (eSenRAF, eSenETO, eSenRep) compared to proliferating (Prolif.) and quiescent (eQuiescent) cells. This decrease of H4-K16Ac contributed to heterochromatin assembly.

**Figure 3:**
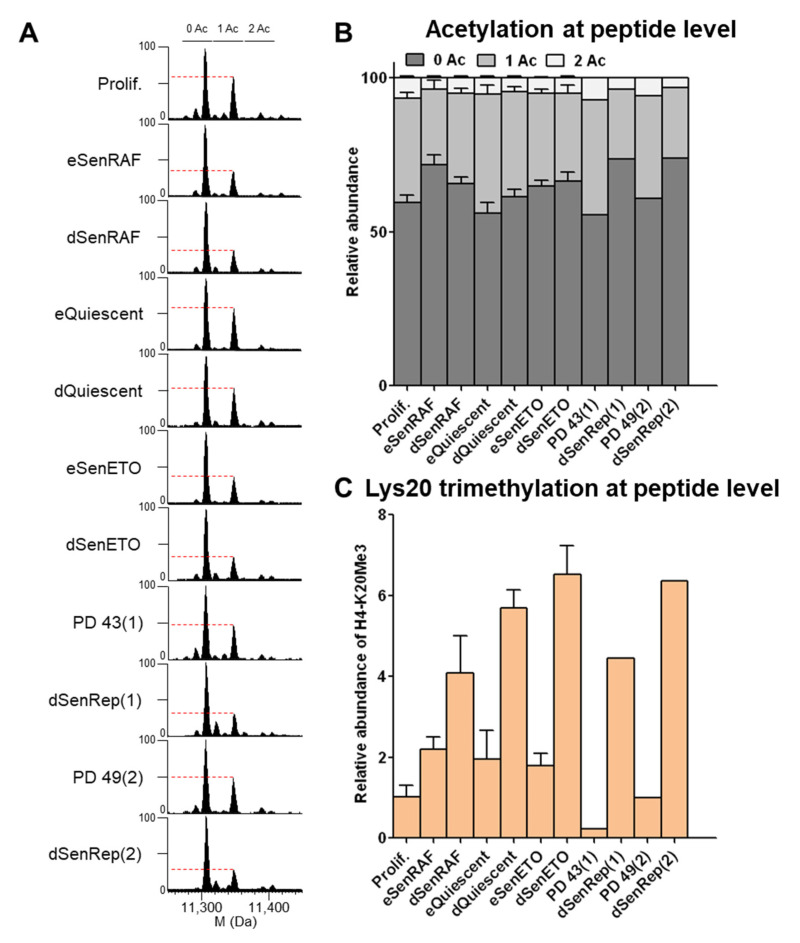
H4 mono-acetylation remained low in deep senescent states and H4-K20Me3 increased progressively with time in conditions of cell cycle arrest. (**A**) Deconvoluted mass spectra of histone H4. The level of the mono-acetylated form is indicated by a dashed bar. (**B**) Relative abundance of H4 acetylated forms quantified on tryptic peptide Gly4-Arg17. (**C**) Relative abundance of H4-K20Me3 quantified on the Lys20-Arg23 peptide; the rest is di-methylated. Experiments were performed on three independent biological replicates. MS/MS spectra corresponding to those H4 peptides are given in Figure S4).

When cells were maintained senescent for 20 days (deep senescence), the levels of H4 mono- and di-acetylation remained low, as observed in early senescent samples (Figure 3A,B), and heterochromatin persisted (Figure S1A). Bottom-up proteomics was used to localize acetylated residues, and it demonstrated that K16 and then K12 were the most prominently acetylated residues (Figure S4). DNA compaction was even more marked when cells were treated with etoposide for a longer time (dSenETO) (Figure S1). However, H4 mono-acetylation level remained higher when cells were quiescent for 20 days (dQuiescent) (Figure 3A,B).

H4-K20Me3 is a repressive histone mark that was reported to increase during the RAS-induced senescence of IMR90 fibroblasts and to participate in transcriptional repres-sion and chromatin compaction [23,24]. H4-K20Me3 was also reported to increase in quiescent fibroblasts [34].

Our analysis showed a progressive increase of H4-K20Me3 (at the peptide level) during both quiescent and senescent proliferative arrests for 5 and 20 days (Figure 3C). H4-K20Me3 increased about 2-fold in cells arrested for 5 days and about 4–6 fold in cells arrested for 20 days compared to proliferating cells. H4-K20Me3 was also increased in senescent cells induced by expression of a dominant negative TRF2 protein for 18 (eSenTRF2D) and 28 days (dSenTRF2D) (Figure S5). In this condition, cells are enlarged, flattened, SA-β-galactosidase activity is increased, and DNA is condensed as for replicatively senescent WI-38 (Figure S1). In addition, replicatively senescent human lung MRC-5 fibroblasts presented the same features (Figure S5). Our results highlight a progressive increase in H4-K20Me3 levels during quiescence and during several types of induced senescence in fibroblasts.

### 3.3. H3.1/2-K27Me2/Me3 and K36Me2 Accumulate with Time in Conditions of Cell Cycle Arrest

H3 histones are highly modified at many distinct sites. The main covalent modifications are lysine methylation (mono-, di-, or tri-methylation) and acetylation [35].

**Figure 4:**
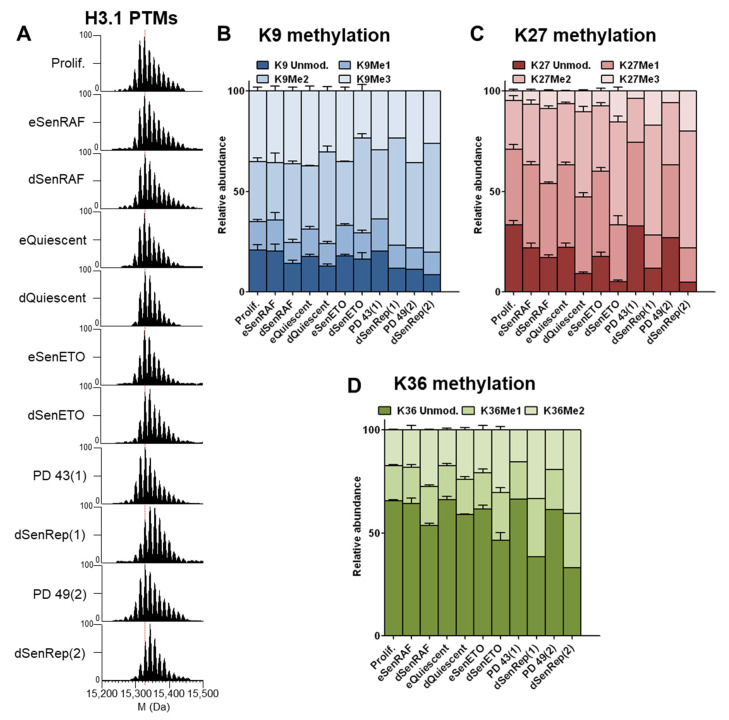
H3.1/2-K27Me2/Me3 and K36Me2 accumulated with time in conditions of cell cycle arrest. (**A**) Deconvoluted mass spectra of histone H3.1. The most abundant peak in prolif. conditions is indicated with a red dashed line. Relative abundance of (**B**) H3.1/H3.2-K9 (Lys9-Arg17 peptide), (**C**) H3.1/2-K27, and (**D**) H3.1/2-K36 methylation isoforms quantified on Lys27-Arg40 peptide. Table S4 summarizes the relative abundancies of the different modified forms of those 2 peptides while the most representative MS/MS spectra are given in Figure S6. Of note, H3K36Me3 was not detected under our conditions, probably due to insufficient analytical sensitivity.

**Figure 5:**
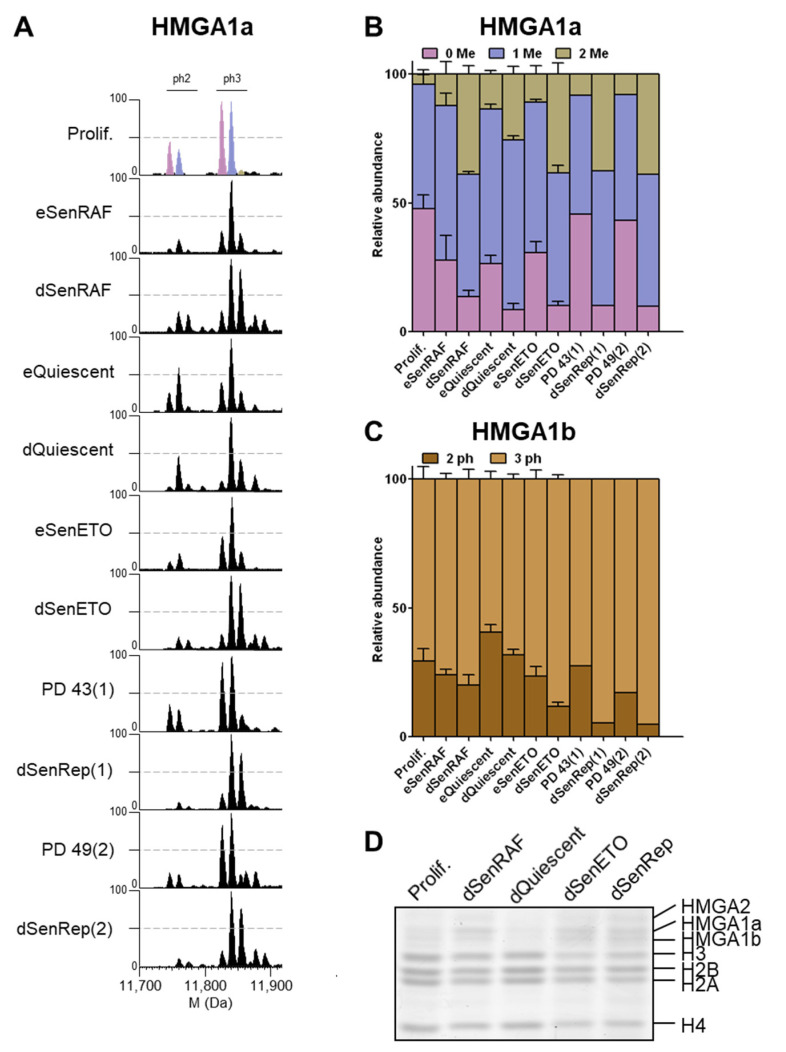
HMGA1a di-methylation and HMGA1b tri-phosphorylation accumulated in deep senescent conditions. (**A**) Deconvoluted mass spectra of HMGA1a isoforms. The colored peaks show 0 (pink), 1 (purple), or 3 (olive) methyl modifications for the di- and tri-phosphorylated forms. The same color scheme is used in B for the quantification. (**B**) Relative abundance of HMGA1a methylation states measured at the protein level. (**C**) Relative abundance of HMGA1b phosphorylation states quantified on intact protein profiles. (**D**) HMGA1 and HMGA2 expression profiles in conditions of long-term cell cycle arrest on SDS-PAGE and Coomassie blue staining. Mass differences were consistent with methylation and phosphorylation differences (14 and 80 Da, respectively).

At the protein level, we noticed that H3.1 (the most abundant H3 variant in WI-38 fibroblasts) was more highly modified by methylation and/or acetylation in non-proliferating cells (early senescence and quiescence) compared to cycling cells (Figure 4A). These modifications seem to accumulate with time, because this phenomenon was exemplified in long-term cell cycle arrest conditions. Analysis at the peptide level revealed that highly methylated forms accumulated predominantly on K27 and K36. The level of H3-K9Me3 remained the same in all conditions, except that it was slightly but reproducibly decreased in deep senescent conditions induced by DNA damage to the benefit of H3-K9Me2 (Figure 5B and Figure S7). We observed a strong and reproducible accumulation of K27Me2/Me3 and K36Me2 with time of cell cycle arrest that could explain what we observe at the protein level (Figure 4C). H3-K27Me2 and H3-K27Me3 accumulated approximately 2 fold and 2–4 fold in long-term cell cycle arrest conditions, respectively. Despite H3-K36Me1 levels remaining globally the same in all conditions, H3-K36Me2 increased in long-term cell cycle arrest by 1.5–2 fold (Figure 4D).

### 3.4. HMGA1a Di-Methylation and HMGA1b Tri-Phosphorylation Accumulated in Deep Senescent Conditions

Although this study was focused on core histones, we also examined HMGA1 proteins that were extracted alongside the histone proteins. The HMGA1 gene encodes 2 isoforms, HMGA1a and HMGA1b, generated by alternative splicing [36]. HMGA1b (95 aa) contains an internal 11 aa deletion relative to HMGA1a (106 aa). Interestingly, we observed potential senescence-associated methylation and phosphorylation modifications of HMGA1. Observed mass differences between HMGA1 isoforms were consistent with methylation and phosphorylation modifications (14 and 80 Da, respectively). Similar HMGA1 modifications were described for B16F10 and H1299 cancer cells induced into senescence by DNA damage [37]. HMGA1a non- and mono-methylated forms are equally present in proliferating samples (Figure 5A,B). However, a massive enrichment of the di-methylated forms occurs in deep senescent conditions (i.e., dSenRAF, dSenETO, dSenRep). The same observations were made for dSenTRF2D and MRC-5 RepSen cells (Figure S8). In early senescent samples, the mono-methylated forms accumulated in a similar fashion as eQuiescent cells (Figure 5A,B). The di-methylated form accumulated by 2–3 fold in deep senescent conditions compared to proliferating cells. In dQuiescent conditions, the di-methylated forms of HMGA1a also accumulated, but to a lesser extent. Previous studies indicated that HMGA1a can be mono- and di-methylated essentially at Arg25 residue, while Ser98, Ser101, and Ser102 are preferentially phosphorylated [37,38,39,40]. The bottom-up strategy used in the present paper involving propionylation and trypsin digestion is not ideally suited to studying Arg25 methylation and potential phosphorylation sites [40]. Therefore, the in-depth study of HMGA1 PTMs would warrant further methodological development and investigation not in the scope of the present study. In contrast, HMGA1b does not seem methylated despite the presence of the Arg25 residue, but is present mainly as di- and tri-phosphorylated forms, presumably at Ser87, Ser90, and Ser91 [39]. We noticed a progressive increase of HMGA1b tri-phosphorylated forms in senescent conditions compared to cycling and quiescent samples. HMGA1b tri-phosphorylation relative abundance reached 70% to 90% depending on deep senescent samples (Figure 5C). This was also the case for dSenTRF2D and MRC5 RepSen conditions (Figure S8). In addition to the modification state, HMGA isoforms were present at higher levels in deep senescent conditions as compared to proliferating and quiescent cells. Altogether, we demonstrated that di-methylated HMGA1a strongly accumulates in deep senescent conditions and HMGA1b tri-phosphorylated form specifically increased in deep senescent conditions. These modifications along with increased HMGA1 protein levels in deep senescent conditions could play an important role in the stable cell cycle arrest and heterochromatin assembly compared to proliferating and dQuiescent cells [27,37].

## 4. Discussion

H2B exists as multiple variants in mammals that differ by a small number of amino acids, but very little is known about their potential physiological and functional specifici-ties. The small number of amino acid differences makes it difficult to distinguish them by chromatographic or electrophoretic methods and no antibodies distinguishing them have yet been described to our knowledge. However, they are distinguishable by their intact mass, so that mass spectrometry is a technique of choice to distinguish them [15,21].

Here, we identified an enrichment of H2B type 1-K at the protein and peptide levels in deep senescent conditions with persistent DNA damages (dSenETO, dSenRep, dSenTRF2D, MRC-5 dSenRep). RT-qPCR experiment showed that total mRNA level of all tested canonical H2B variants decreased strongly in early (5 days) and deep (20 days) conditions of cell cycle arrest compared to cycling cells. This is due to the fact that during DNA replication, canonical histone mRNAs are induced about 30 fold to package newly synthesized DNA [12]. However, we noticed an increase of polyA mRNA level for all tested H2B variants in cell cycle arrested conditions. This can be explained by a basal expression of histone genes even when cells do not proliferate and by the absence of SLBP, leading to the accumulation of stable histone polyA mRNAs for those H2B genes that contain a polyadenylation site downstream of their stem-loop sequence [13,14,15,16]. We identified an increase of H2B type 1-K specifically in deep senescent conditions with persistent DNA damages despite an increase of all H2B polyA mRNA levels. Hence, under these conditions, H2B type 1-K must be regulated post-transcriptionally, such as by: (i) increased mRNA export to the cytoplasm, (ii) increased export of the gene product to the nucleus, (iii) increased deposition, or (iv) decreased eviction/degradation. This regulation might be linked to the ATM/ATR kinases pathways that are activated by DNA damage. H2B type 1-K enrichment in senescence parallels the increase in the H2A variant H2A.J that we previously described [22]. Since H2A forms heterodimers with H2B, it is likely that H2A.J-H2B-type 1-K heterodimers increase specifically in senescent cells. Several histone chaperones for H2A/H2B have been described including the NAP family chaperones and the FACT complex. Intriguingly however, the *HIST1H2B1K* gene encoding H2B type 1-K was first identified in a 2-hybrid screen with HIRA, a subunit of a histone chaperone complex for the H3.3-H4 histones. In this study, HIRA was shown to interact with both H2B type 1-K and H4 [41]. HIRA has important roles in the chromatin dynamics of senescent cells [14]. The functional importance of the HIRA-H2B type 1-K merits further study as it is not rare for histone chaperones to interact with multiple histone types whilst facilitating chromatin dynamics [42].

H2B type 1-K is discernable from most other H2B variants by containing an Ala instead of a Ser at position 124 near the C-terminus in a region that should be accessible outside of the nucleosome. This substitution of a phosphorylatable residue to a non-phosphorylatable one could have important functional effects, although Ser-124 phosphorylation of H2B has not yet been reported. Further genetic experiments will be necessary to determine whether H2B type 1-K has specific functional roles in senescence.

In a previous paper, we identified a senescent-specific deacetylation of H4-K16Ac that occurs early in the establishment of senescence and that contributes to heterochromatin assembly. Here, we noticed that the decreased acetylation on H4 was maintained in deep senescent conditions concordant with a maintained heterochromatin assembly [28].

In this study, we also highlighted an increase with time of H4-K20Me3, H3.1/2-K27Me2/Me3, and H3.1/2-K36Me2 in all types of post-mitotic fibroblasts in vitro. Hence, these histone variants and marks accumulated independently of the senescent state and DNA damages. Interestingly, H4-K20Me3 levels were found to increase progressively with age in the rat liver and kidney [43]. Likewise, increases of H3-K27Me3 were observed in quiescent muscle stem cells during aging [44]. Furthermore, H3.3-K27Me2 and H3.3-K36Me2 were found to accumulate during aging in mouse liver, kidney, brain, and heart [45]. The molecular basis for the aging-associated accumulation of these specific H3 and H4 methylation marks has not been determined, but they are likely to affect both gene expression and chromatin compaction during the aging of post-mitotic tissues. The recapitulation of their accumulation in post-mitotic fibroblasts in vitro should facilitate the study of their regulation.

In addition to histone variants and PTMs, we examined HMGA1 isoforms. Similarly to what has been observed by Tran et al. for cancer cells (B16F10 and H1299) induced into senescence by DNA damage [37], we noticed a strong increase of di-methylated HMGA1a, and total HMGA1a/b protein levels, with time in all deep senescent states compared to cycling cells. These modifications are thus characteristic of senescence for both normal and cancer cells. The di-methylated forms also accumulate to a lesser extent in quiescent cells, with no visible increase in HMGA1 protein levels in quiescence. In senescent WI-38 cells, HMGA1b is less overexpressed than HMGA1a. We also noticed a modest but significant increase of the HMGA1b tri-phosphorylated form in deep senescent states. Altogether, the quantity and modification state of HMGA1 proteins could be important for senescence maintenance. Antibodies to specific methylated and phosphorylated sites of histones have proven their effectiveness for detecting and characterizing these modifications. We propose that the development of antibodies to di-methylated HMGA1a (dimethylation presumably occurs at Arg25 [37,39]) and tri-phosphorylated HMGA1b will be useful in specifically identifying senescent cells and in studying the regulation and the function of these modifications.

## Supplementary Materials

The following are available online at https://www.mdpi.com/article/10.3390/proteomes9020030/s1, Table S1: Primers used in qPCR experiments in 5′ to 3′ orientation; Table S2: Masses and accuracies of histone H2B variants observed by UHPLC-MS as found in a dSenRep sample; Table S3: Comparison of protein sequences of studied H2B variants; Table S4: Different modified forms of histone H3 Lys9-Arg17 and Lys27-Arg40 peptides presenting with relative abundancies >1%; Figure S1: Heterochromatin, cell cycle arrest, and γH2A.X foci in proliferative, quiescent, and senescent conditions; Figure S2: H2B type 1-K is specifically enriched in deep senescent cells with persistent DNA damage (related to Figure 2); Figure S3: MS/MS spectra of histone H2B-specific peptides; Figure S4. Histone H4 acetylation and methylation levels; Figure S5: H4 acetylation is low in deep senescent conditions and H4-K20Me3 accumulates with time in long-term cell cycle arrest conditions (related to Figure 3); Figure S6: Histone H3.1/2 methylation states; Figure S7: H3.1/2-K27Me2/Me3 and K36Me2 accumulate with time in conditions of cell cycle arrest (related to Figure 4); Figure S8: HMGA1a di-methylation and HMGA1b tri-phosphorylation accumulate in deep senescent conditions (related to Figure 5).
