# Supplementary material for: H2B Type 1-K Accumulates in Senescent Fibroblasts with Persistent DNA Damage along with Methylated and Phosphorylated Forms of HMGA1
Source: Proteomes. 2021 Jun 21;9(2):30. doi: 10.3390/proteomes9020030 (PMC8293446; doi:10.3390/proteomes9020030)
Supplement: Supplementary file 1 [file proteomes-09-00030-s001.zip › proteomes-1250046-SI.pdf]

Supplemental Information

H2B Type 1-K Accumulates in Senescent Fibroblasts with Persistent DNA Damage along with Methylated and Phosphorylated Forms of HMGA1

Kévin Contrepois [1,2,3], Carl Mann [1,*] and François Fenaille [4,*]

[1] Institute for Integrative Biology of the Cell (I2BC), Université Paris-Saclay, CEA, CNRS, 91190 Gif-sur-Yvette, France; kcontrep@stanford.edu
[2] Department of Genetics, Stanford University School of Medicine, Stanford, CA 94305, USA;
[3] Stanford Cardiovascular Institute, Stanford University, Stanford, CA 94305, USA
[4] Université Paris-Saclay, CEA, INRAE, Département Médicaments et Technologies pour la Santé (DMTS), MetaboHUB, F-91191 Gif-sur-Yvette, France
* Correspondence: carl.mann@cea.fr (C.M.); francois.fenaille@cea.fr (F.F.); Tel.: +33-1-69-08-92-94 (C.M.); +33-1-69-08-79-54 (F.F.)

Table S1. Primers used in qPCR experiments in 5′ to 3′ orientation. All the primers were designed using Primer3 and BLAST: http://www.ncbi.nlm.nih.gov/tools/primer-blast.

Name	Sequence
HIST1H2BK_Total_F	TCGTAGTTCGCCTTCAACATGCCG
HIST1H2BK_Total_R	CGGCTTTCTTCGAGCCCTTCTTGG
HIST1H2BK_PolyA_F	TGCCAAGGAGGGACTTTCTCTGGA
HIST1H2BK_PolyA_R	AGAGGCCAGCTTTAGCTTGTGGA
HIST1H2BH_Total_F	TGATCCAGCTAAGTCCGCTCCCG
HIST1H2BH_Total_R	ATGGCTTTGGAGGAGATGCCGGT
HIST1H2BD_Total_F	AACGCTACGATGCCTGAACCTACCA
HIST1H2BD_Total_R	CTTGCGCTTCTTCCCGTCCTTCTTC
HIST1H2BD_PolyA_F	TCCTGGTTTGCTGCACACTGGT
HIST1H2BD_PolyA_R	ACACCTTTAGTTCCTTCCCCTCGGT

Table S2. Masses and accuracies of histone H2B variants observed by UHPLC-MS as found in a dSenRep sample. H2B spectra were internally recalibrated using H4-derived ions. Theoretical and observed masses are monoisotopic masses. Theoretical masses were calculated based on known primary sequences and PTMs.

Variant (UniprotKB recommended names)	Genes	UniprotKB Accession	Theoretical Masses (Da)	Observed Masses (Da)	Δm	Error (ppm)
Histone H2B type 1-K	HIST1H2BK	O60814	13,750.52	13,750.50	−0.02	−1.5
Histone H2B type 1-H*	HIST1H2BH	Q93079	13,752.50	-	-	-
Histone H2B type 1-C/E/F/G/I	HIST1H2BC/E/F/G/I	P62807	13,766.52	13,766.53	0.01	0.7
Histone H2B type 2-E	HIST2H2BE	Q16778	13,780.53	13,780.50	−0.03	−2.2
Histone H2B type 2-F	HIST2H2BF	Q5QNW6	13,780.53	13,780.50	−0.03	−2.2
Histone H2B type 1-D	HIST1H2BD	P58876	13,796.53	13,796.51	−0.02	−1.4
Histone H2B type 1-B	HIST1H2BB	P33778	13,810.54	13,810.52	−0.02	−1.4
Histone H2B type 1-M	HIST1H2BM	Q99879	13,849.59	13,849.44	−0.15	−10.8

*: Isotope massifs of H2B type 1-K and type 1-H overlap, thus it is almost impossible to derive a precise mass for H2B type 1-H.

Table S3. Comparison of protein sequences of studied H2B variants.

Variants	Varied Amino Acid Positions					
	**2–4**	**9**	**18–19**	**21**	**38**	**124**
HIST1H2BK	EPA	A	VT	A	V	A
HIST1H2BH	DPA	A	VT	A	V	S
HIST1H2BC/E/F/G/I	EPA	A	VT	A	V	S
HIST2H2BE	EPA	A	VT	A	I	S
HIST2H2BF	DPA	A	VT	V	V	S
HIST1H2BD	EPT	A	VT	A	V	S
HIST1H2BB	EPS	A	IT	A	I	S
HIST1H2BM	EPV	V	IN	A	V	S

Table S4. Different modified forms of histone H3 Lys9-Arg17 and Lys27Arg40 peptides presenting with relative abundancies >1%.

			**Prolif.**	**eSenRAF**	**dSenRAF**	**eQuiescent**	**dQuiescent**	**eSenETO**	**dSenETO**	**PD 43(1)**	**dSenRep(1)**	**PD 49(1)**	**dSenRep(2)**
**Peptide** H3-K_9STGGK_{14}APR	**RT (min)**	**$[M+2H]^{2+}$**	**Approx. %**	**Approx. %**	**Approx. %**	**Approx. %**	**Approx. %**	**Approx. %**	**Approx. %**	**Approx. %**	**Approx. %**	**Approx. %**	**Approx. %**
Unmod.	31.30	535.3037	15.3	15.3	10.8	12.7	9.9	13.4	13.4	14.8	8.8	8.0	7.5
K9 Me1 + K14 Ac	33.27	535.3037	4.6	4.8	2.3	3.6	2.4	3.7	2.3	4.9	2.8	2.6	1.6
K9 Me1	35.07	542.3115	9.5	10.7	8.0	10.1	8.8	11.5	10.6	11.0	8.7	7.9	9.4
K9 Me2	24.18	521.3062	24.4	22.8	33.1	23.9	39.2	25.2	41.5	27.6	43.3	33.9	48.5
K9 Me3 + K14 Ac	22.39	521.3062	4.5	3.1	2.0	3.3	1.1	3.0	0.4	4.5	2.8	4.5	1.9
K9 Me3	24.05	528.3140	30.7	32.5	34.3	33.9	29.1	32.2	23.0	24.7	20.6	31.1	24.1
K14 Ac	29.57	528.2958	5.7	5.1	3.5	4.9	3.0	4.5	2.9	5.5	3.0	3.4	1.1
K9 Me2 + K14 Ac	22.56	514.2984	5.3	5.8	6.0	7.5	6.5	6.5	5.8	6.8	9.9	8.7	5.8
			Prolif.	eSenRAF	dSenRAF	eQuiescent	dQuiescent	eSenETO	dSenETO	PD 43(1)	dSenRep(1)	PD 49(1)	dSenRep(2)
**Peptide** H3-K_{27}SAPATGGVK_{36}KPHR	**RT (min)**	**$[M+2H]^{2+}$**	**Approx. %**	**Approx. %**	**Approx. %**	**Approx. %**	**Approx. %**	**Approx. %**	**Approx. %**	**Approx. %**	**Approx. %**	**Approx. %**	**Approx. %**
Unmodified	38.06	829.47285	26.1	18.5	13.5	17.2	6.8	12.3	3.0	25.3	1.5	19.0	1.1
K27 Me1	40.95	836.48067	23.5	26.6	22.0	27.4	25.3	28.2	16.1	27.1	8.2	22.1	6.3
K36 Me1	40.16	836.48067	3.7	1.4	1.0	2.3	0.4	2.5	0.0	4.1	0.0	4.6	0.3
K27 Me2	32.71	815.47539	13.6	15.6	14.5	17.0	21.4	16.1	21.8	11.7	22.6	17.1	19.7
K36 Me2	34.69	815.47539	5.0	2.1	2.5	2.8	1.8	2.8	2.0	3.5	10.2	3.5	3.5
K27Me3	32.61	822.48321	3.0	3.6	3.5	4.8	5.4	4.9	5.6	2.4	6.2	3.1	6.2
K27Me2 + K36 Me1	34.69	822.48321	7.1	8.9	10.5	8.3	9.9	9.7	13.2	6.7	17.3	8.6	16.7
K27 Me1 + K36 Me2	37.50	822.48321	8.2	9.2	10.9	8.8	9.3	10.0	8.4	8.2	5.1	9.4	8.5
K27 Me3 + K36 Me1	34.60	829.49104	0.8	2.0	3.4	1.0	3.4	1.4	6.1	0.9	7.5	1.9	7.2
K27 Me1 + K36 Me1	43.03	843.4885	5.5	5.4	4.1	4.9	3.5	4.2	3.7	6.4	3.4	4.6	2.2
K27 Me3 + K36 Me2	29.64	808.48576	0.4	1.0	1.8	0.7	1.5	1.0	3.8	0.4	3.3	0.8	6.5
K27 Me2 + K36 Me2	29.64	801.47793	3.1	5.7	12.3	4.9	11.3	6.9	16.2	3.4	14.7	5.4	21.8

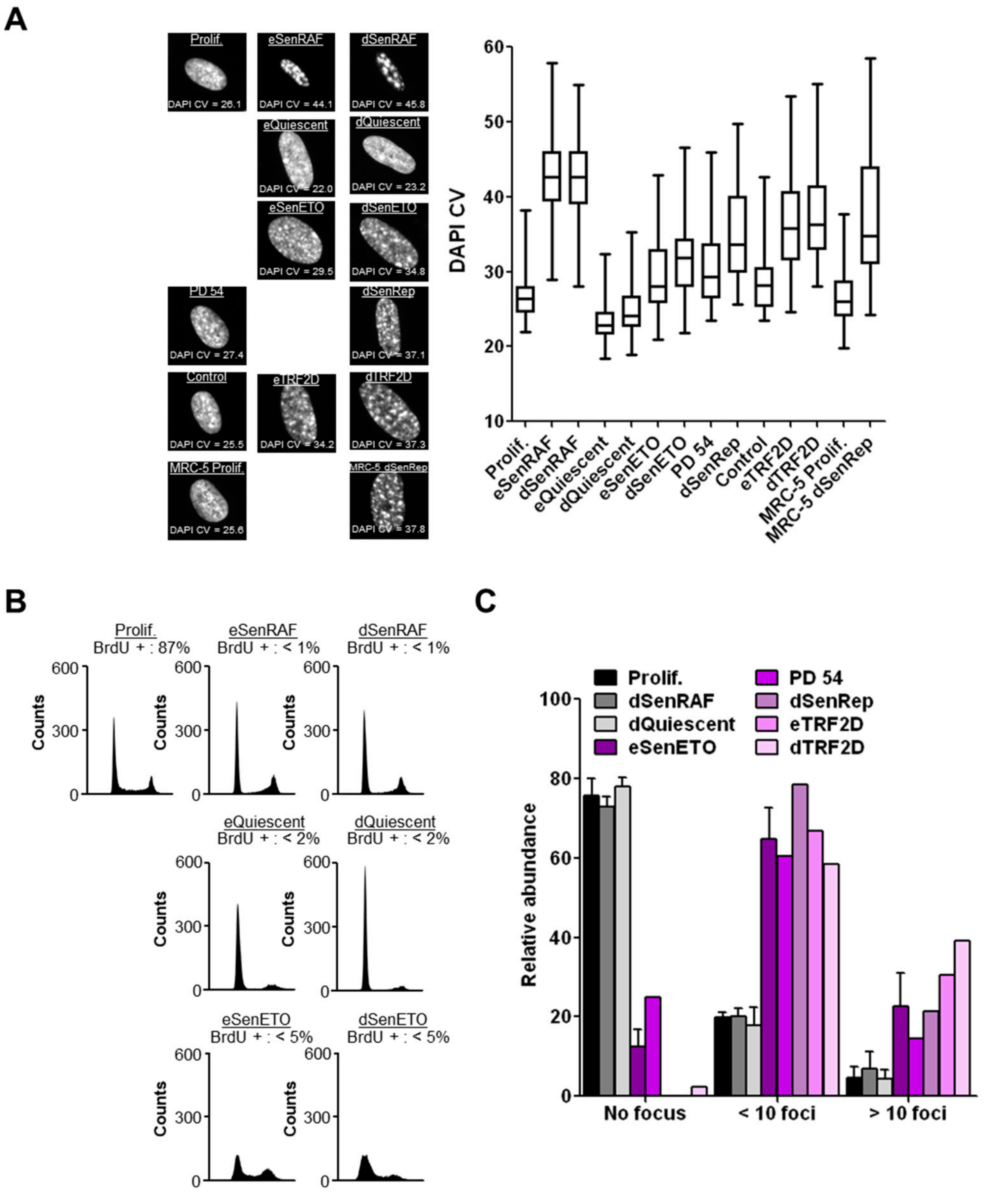

Figure S1. Heterochromatin, cell cycle arrest and γH2A.X foci in proliferative, quiescent and senescent conditions. (**A**) DNA DAPI staining of representative nuclei and boxplots of DAPI CV (n > 60 nuclei per conditions). (**B**) DNA content by flow cytometry. The percentage of cells incorporating BrdU after 24 hours of incubation is indicated. (**C**) Quantification of the number of γH2A.X foci per nucleus. Error bars represent at least 200 nuclei from at least three independent biological replicates.

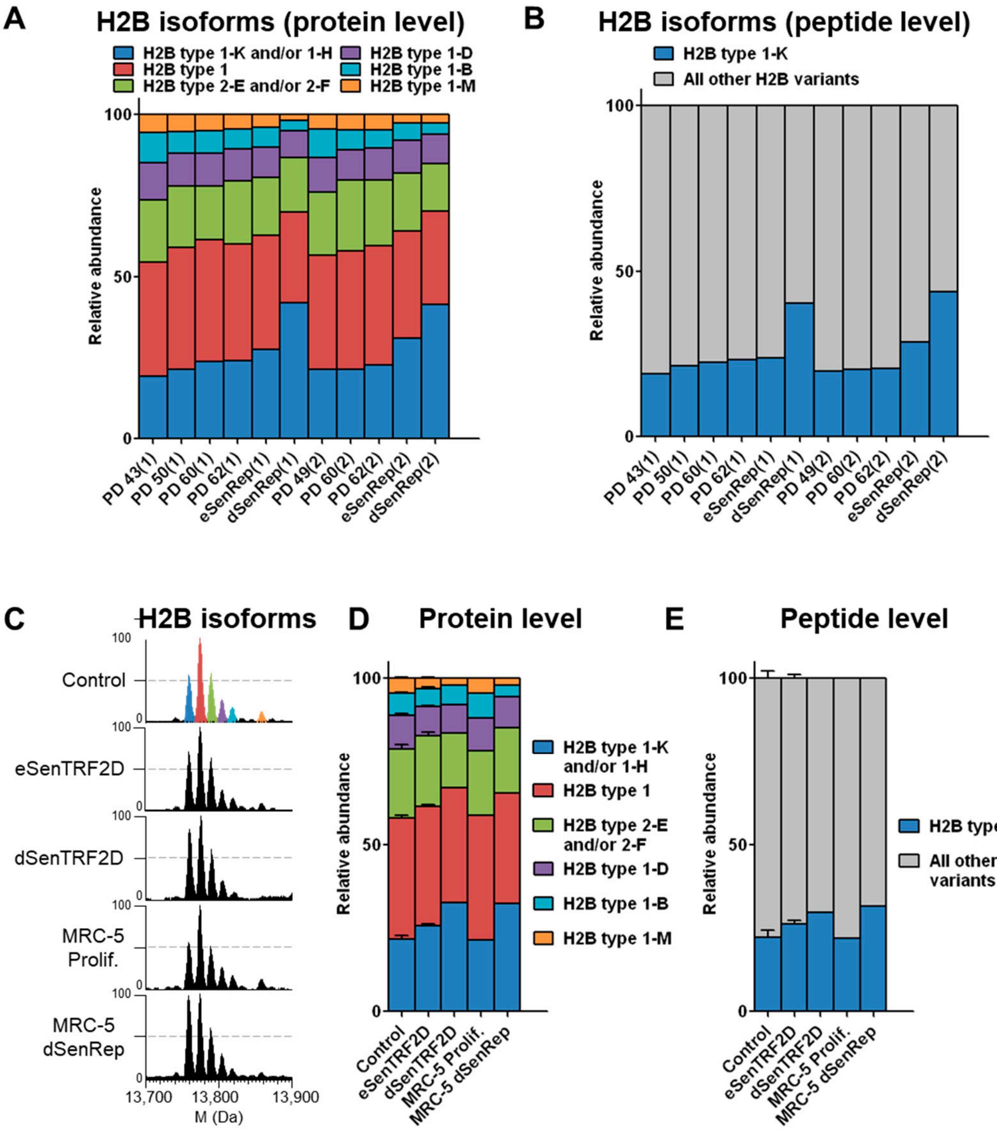

Figure S2. H2B type 1-K is specifically enriched in deep senescent cells with persistent DNA damage (related to Figure 2). (**A**) Relative abundance of H2B isoforms during two independent kinetics of replicative senescence at the protein and (**B**) peptide (Leu100- Lys125) levels. (**C**) Deconvoluted mass spectra of histone H2B isoforms and (**D**) corresponding relative abundances at the protein and (**E**) peptide (Leu100- Lys125) levels. Experiments were performed on two independent biological replicates.

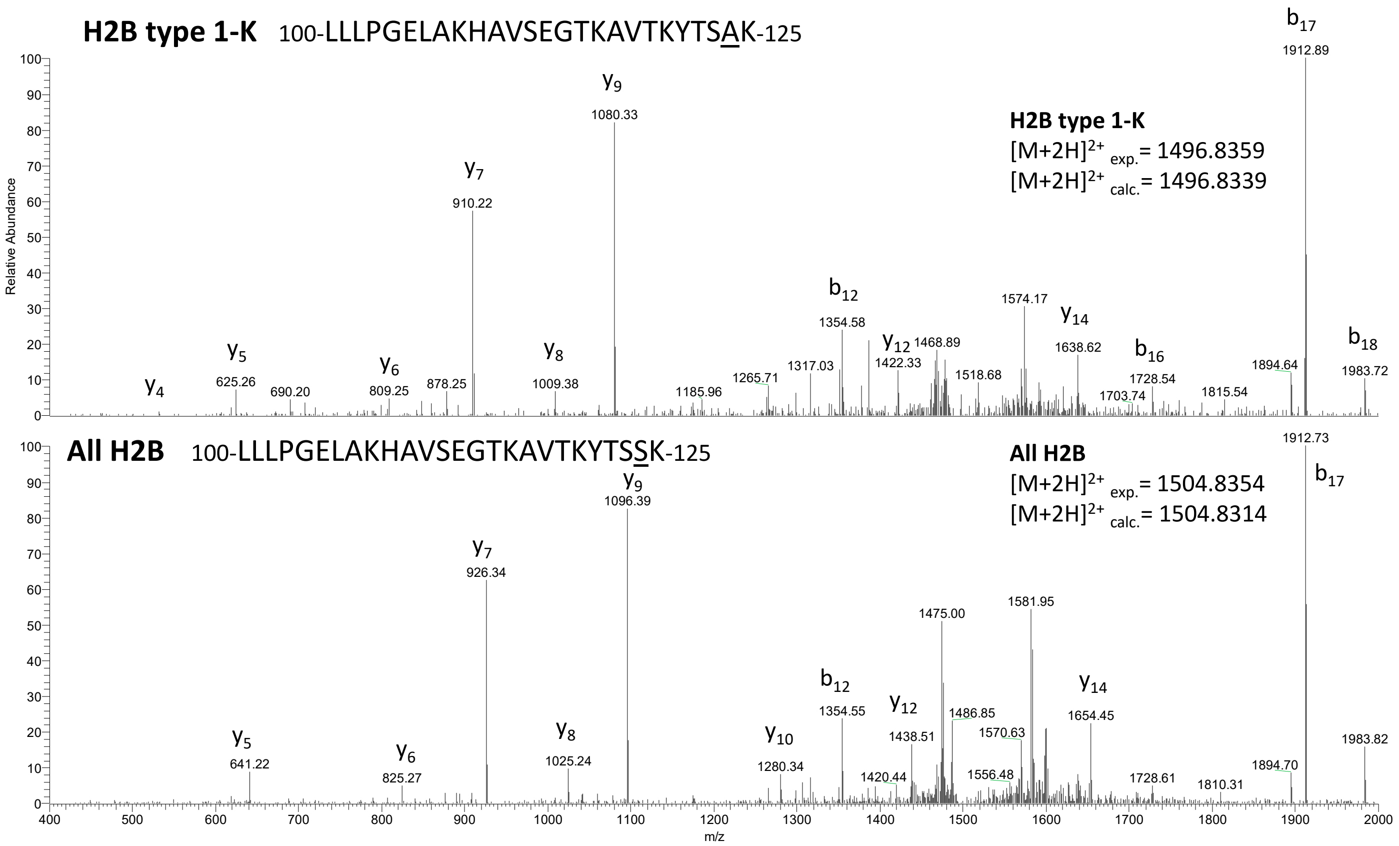

Figure S3. MS/MS spectra of histone H2B-specific peptides. (**A**) H2B type 1-K specific and (**B**) canonic H2B Leu100-Lys125 peptides as found in a dSenRep sample. The most intense ions are labeled. Two rounds of propionylation were performed before and after trypsin digestion to propionylate free unmodified lysines and N-termini.

A). H4 4-GKGGKGLGK$_{12}$GGAK$_{16}$R-17

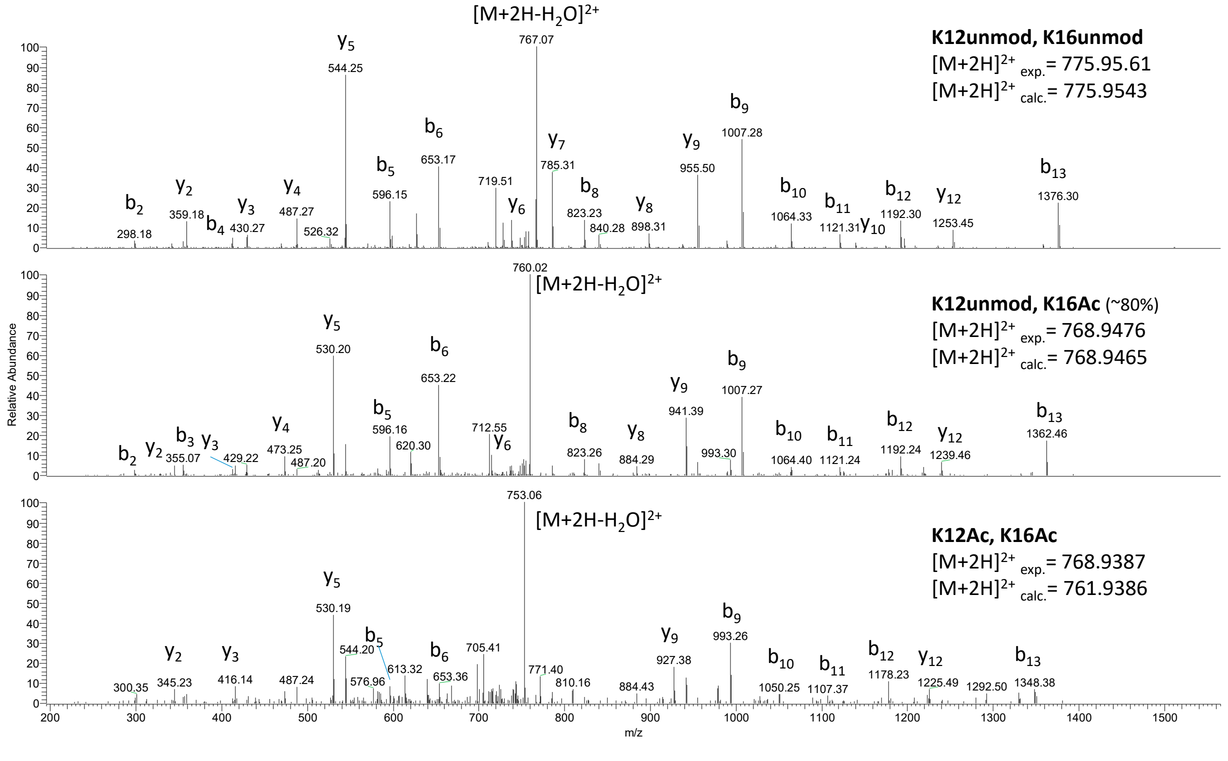

B). H4 20-K_{20}VLR-23

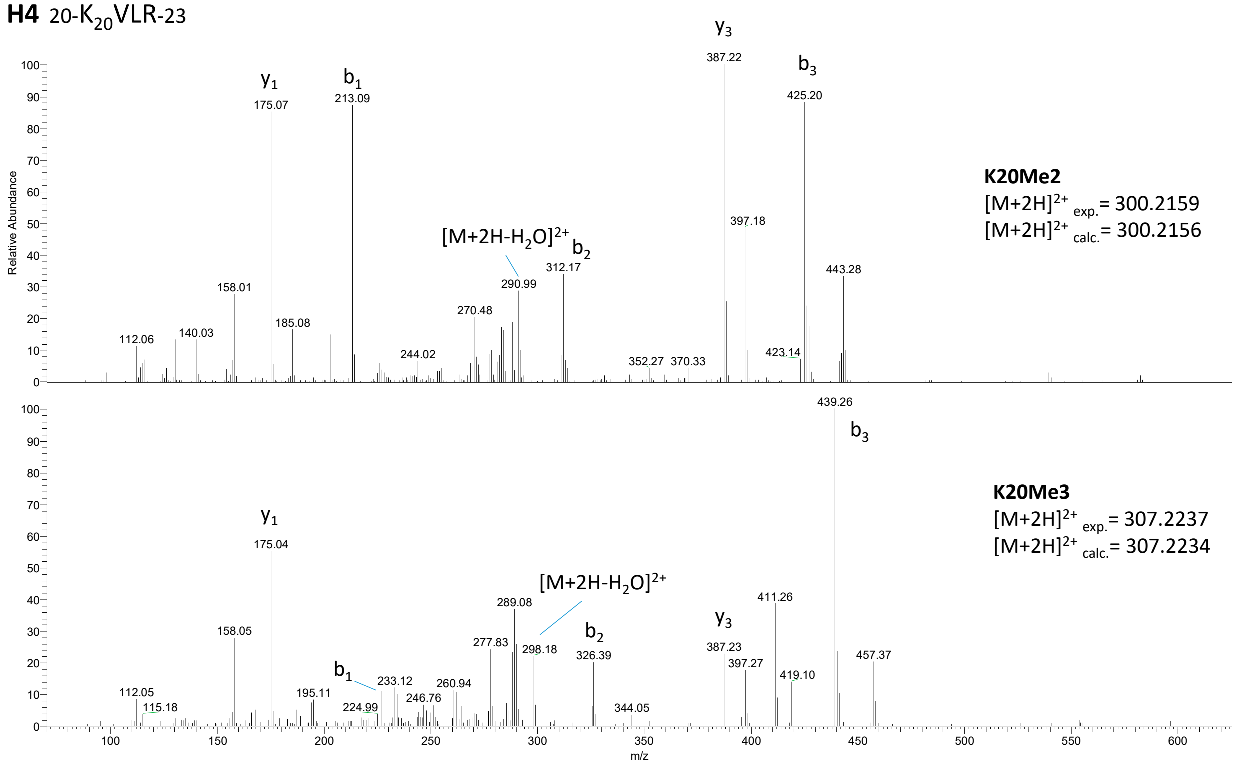

Figure S4. Histone H4 acetylation and methylation levels. MS/MS spectra of (**A**) the unmodified, mono-acetylated and diacetylated H4 tryptic peptide Gly4-Arg17 as found in a dSenRep sample; (**B**) the dimethylated and trimethylated Lys20-Arg23 peptide as found in a dSenRep sample. Two rounds of propionylation were performed before and after trypsin digestion to propionylate free unmodified lysines and N-termini. Consequently, the 14 Da differences observed on particular fragment ions represent the mass difference between acetylated and propionylated lysine residues.

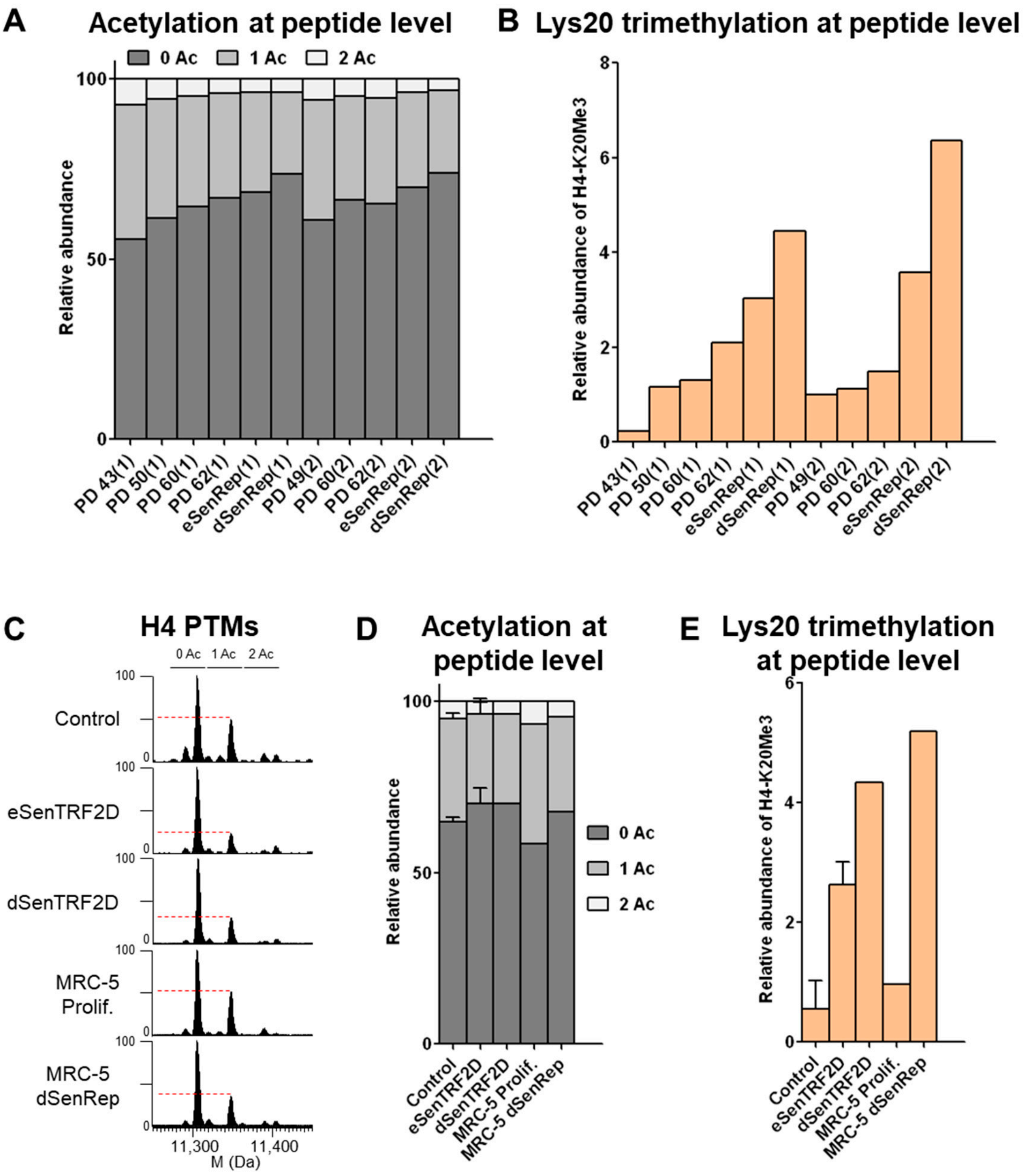

Figure S5. H4 acetylation is low in deep senescent conditions and H4-K20Me3 accumulates with time in long-term cell cycle arrest conditions (related to Figure 3). (**A**) Relative abundance of H4 acetylation and of (**B**) H4-K20Me3 quantified on Gly4-Arg17 and Lys20-Arg23 peptides respectively during two independent kinetics of replicative senescence. (**C**) Deconvoluted mass spectra of histone H4. The level of the most abundant mono-acetylated form is indicated by a dashed bar. (**D**) Relative abundance of H4 acetylation and of (**E**) H4-K20Me3 quantified on Gly4-Arg17 and Lys20-Arg23 peptides respectively. Experiments were performed on two independent biological replicates.

A). H3 9-K_9STGGK_{14}APR-17

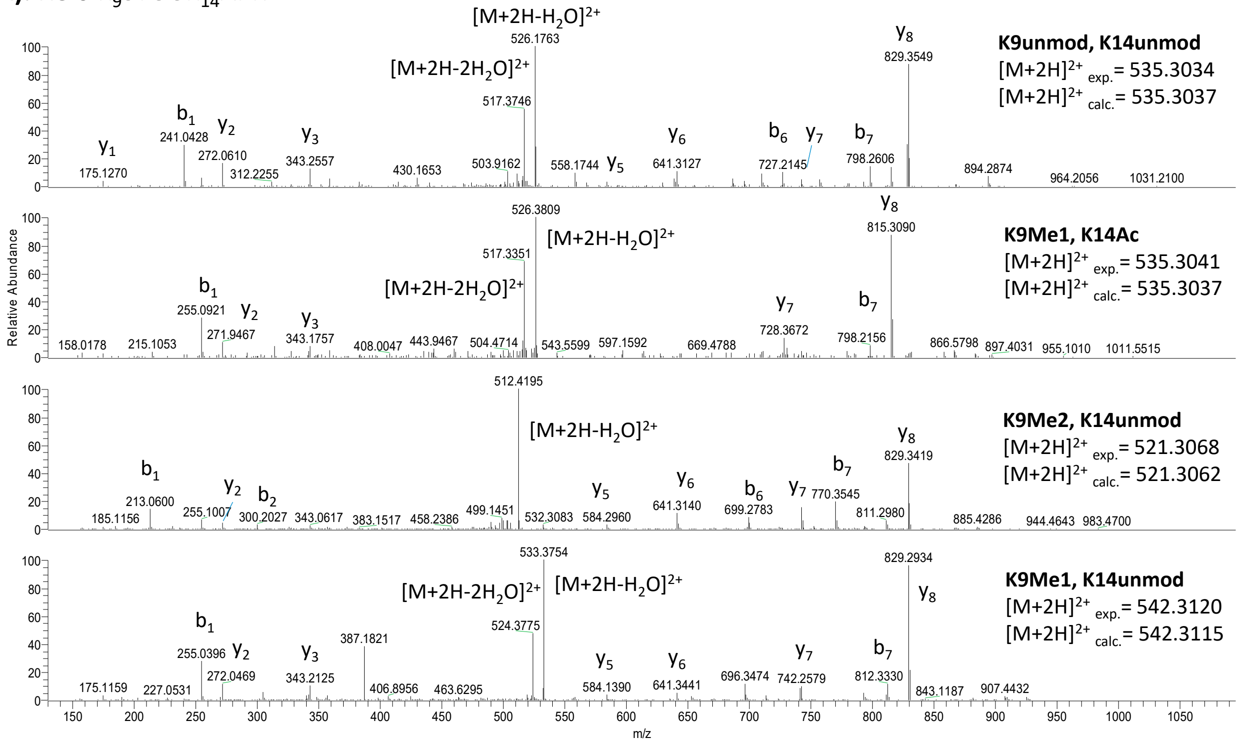

B). H3 27-K_{27}SAPATGGVK_{36}KPHR-40

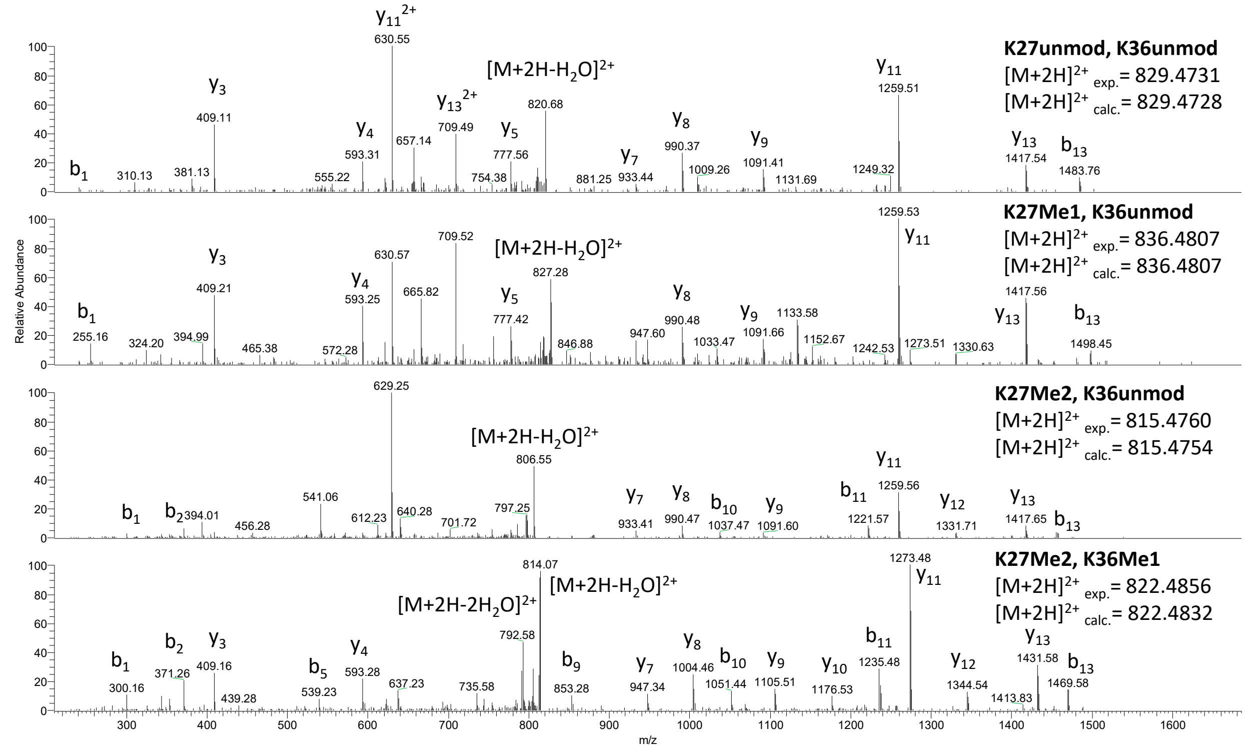

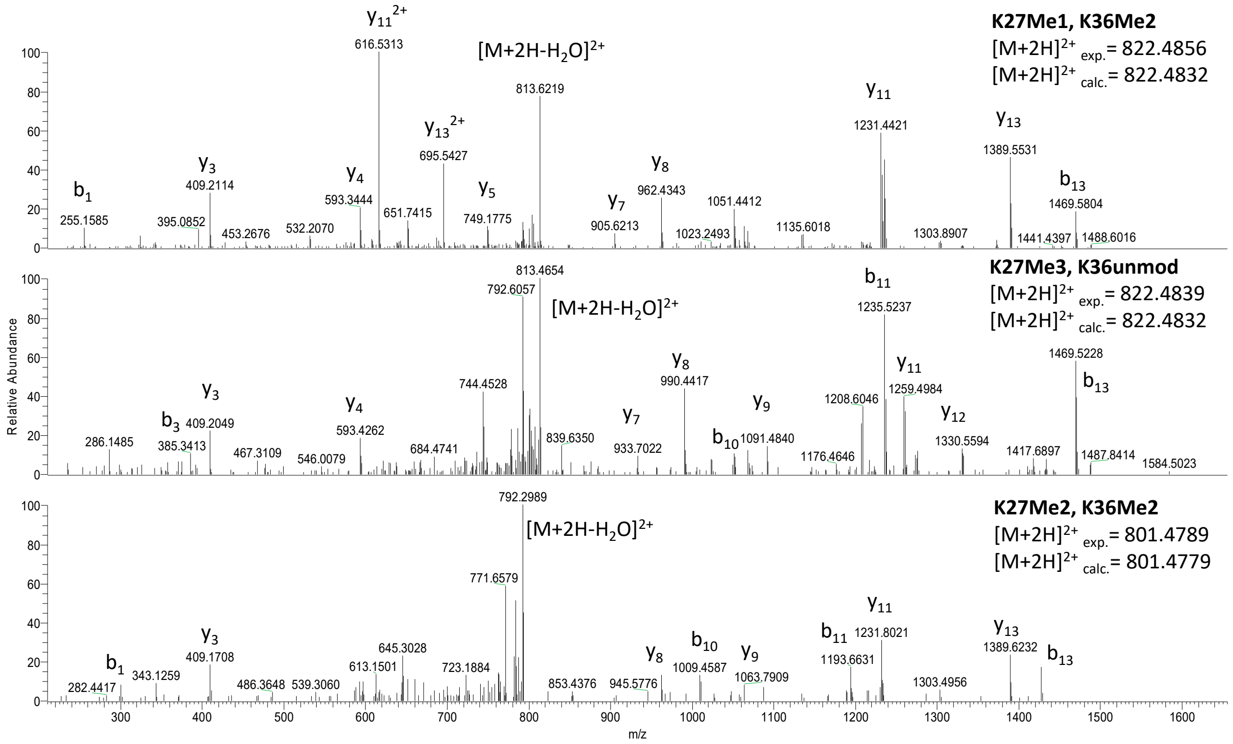

Figure S6. Histone H3.1/2 methylation states. MS/MS spectra of (**A**) the peptides unmodified and methylated at Lys9 as found in a dSenRep sample; (**B**) the peptides unmodified and methylated at Lys27 and Lys36 as found in a dSenRep sample. Those MS/MS spectra are those of the most representative modified peptides detected. Two rounds of propionylation were performed before and after trypsin digestion to propionylate free unmodified lysines and N-termini. Consequently, the 14 Da differences observed on particular fragment ions represent the mass difference between acetylated and propionylated lysine residues.

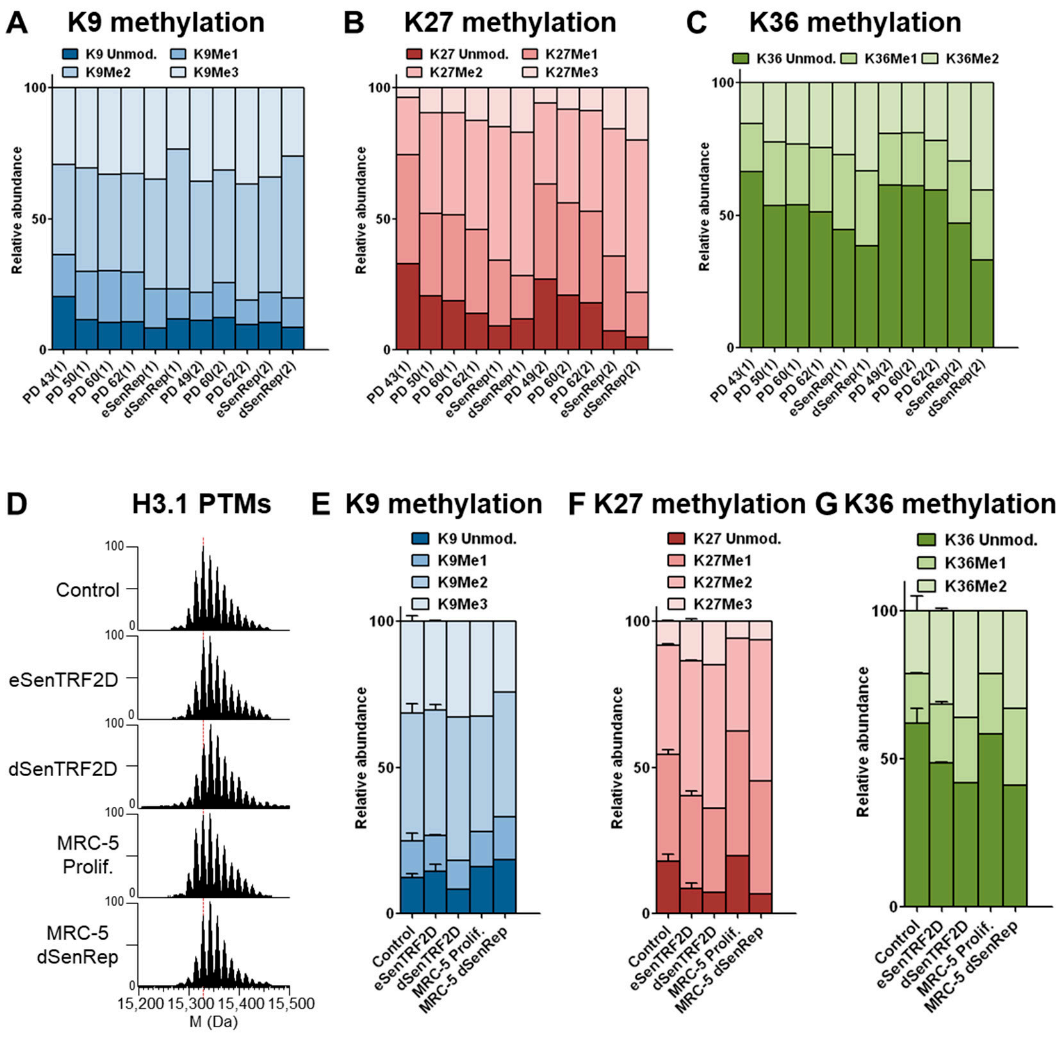

Figure S7. H3.1/2-K27Me2/Me3 and K36Me2 accumulate with time in conditions of cell cycle arrest (related to Figure 4). Relative abundance of (**A**) H3.1/2-K9 (Lys9-Arg17 peptide), (**B**) H3.1/2-K27 and (**C**) H3.1/2-K36 methylation isoforms quantified on Lys27-Arg40 peptide during two independent kinetics of replicative senescence. (**D**) Deconvoluted mass spectra of histone H3.1 isoforms and relative abundance of (**E**) H3.1/2-K9 (Lys9-Arg17 peptide), (**F**) H3.1/2-K27 and (**G**) H3.1/2-K36 methylation isoforms quantified on Lys27-Arg40 peptide. Experiments were performed on two independent biological replicates.

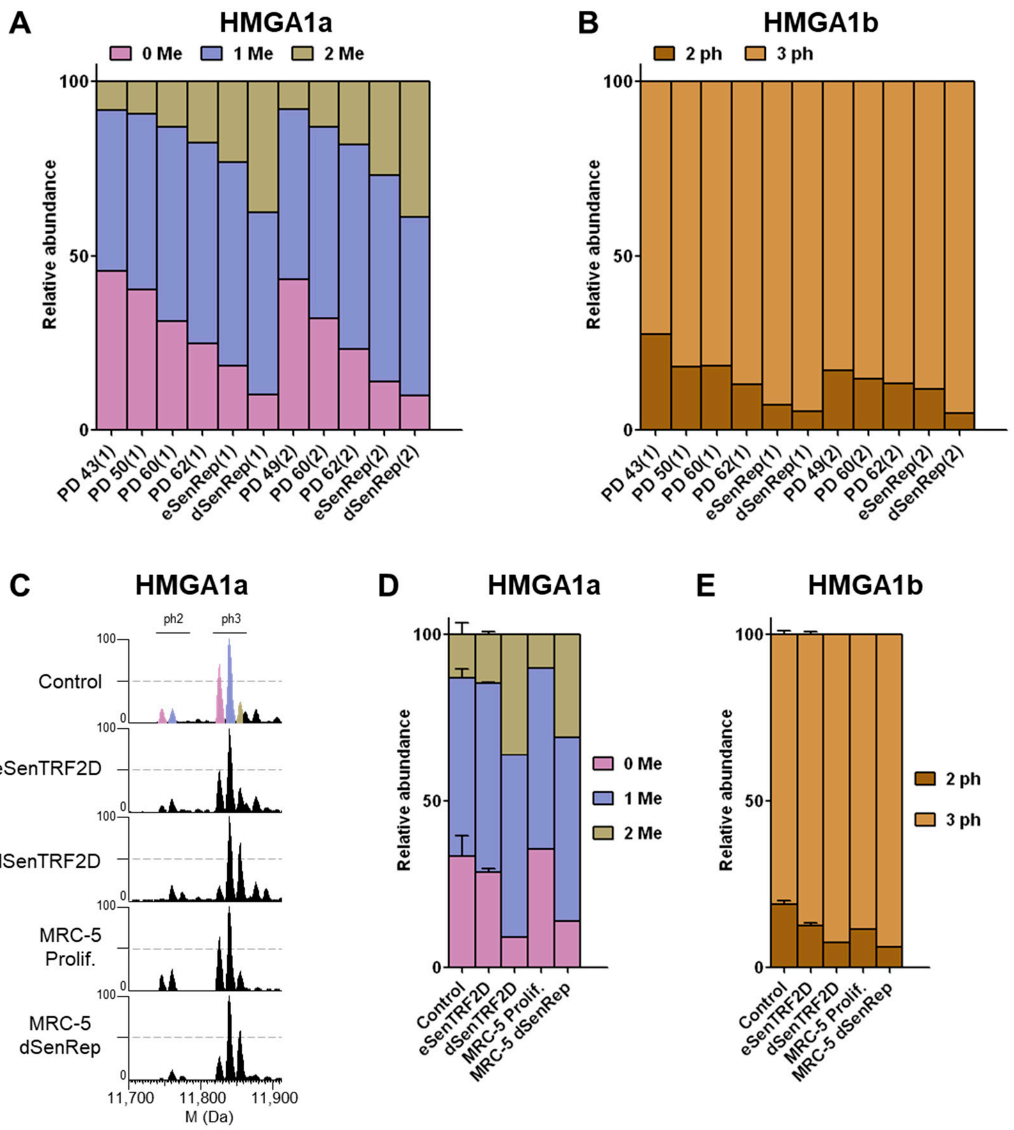

Figure S8. HMGA1a di-methylation and HMGA1b tri-phosphorylation accumulate in deep senescent conditions (related to Figure 5). (**A**) Relative abundance of HMGA1a methylation and (**B**) HMGA1b phosphorylation states during two independent kinetics of replicative senescence. (**C**) Deconvoluted mass spectra of HMGA1a isoforms and (**D**) corresponding relative abundances of methylation states. (**E**) Semi-quantification of HMGA1b phosphorylation states. Experiments were performed on two independent biological replicates.
